# The Prevalence and Characterization of Extended-Spectrum β-Lactamase- and Carbapenemase-Producing Bacteria from Hospital Sewage, Treated Effluents and Receiving Rivers

**DOI:** 10.3390/ijerph17041183

**Published:** 2020-02-13

**Authors:** Luhua Zhang, Xinyue Ma, Li Luo, Nan Hu, Jiayao Duan, Zhongjian Tang, Rujie Zhong, Ying Li

**Affiliations:** 1 Department of Pathogenic Biology, School of Basic Medical Sciences, Southwest Medical University, Luzhou 646000, China; zhluhua@swmu.edu.cn (L.Z.); h20200210@126.com (N.H.); d991806@163.com (J.D.); tt403921424@163.com (Z.T.); Evenlee1987@163.com (R.Z.); 2 Department of Immunology, School of Basic Medical Sciences, Southwest Medical University, Luzhou 646000, China; HMM151126@126.com (X.M.); chenxishiwei@126.com (L.L.)

**Keywords:** carbapenemase, *bla*_NDM_, *bla*_KPC_, hospital sewage, *Enterobacteriaceae*

## Abstract

Hospital sewage plays a key role in the dissemination of antibiotic-resistant genes (ARGs) by serving as an environmental antimicrobial resistance reservoir. In this study, we aimed to characterize the cephalosporin- and carbapenem-resistant isolates from hospital sewage and receiving rivers. The results showed that ESBL (*bla*_CTX-M_) and carbapenemase genes (*bla*_NDM_ and *bla*_KPC_) were widely detected in a number of different bacterial species. These resistance genes were mainly harbored in *Enterobacteriaceae*, followed by *Acinetobacter* and *Aeromonas* isolates. More attention should be given to these bacteria as important vectors of ARGs in the environment. Furthermore, we showed that the multidrug resistance phenotype was highly prevalent, which was found in 85.5% *Enterobacteriaceae* and 75% *Acinetobacter* strains. Notably, the presence of carbapenemase genes in isolates from treated effluents and receiving rivers indicates that the discharges of wastewater treatment plants could be an important source for high-risk resistance genes propagation to the environment. In conclusion, this study shows a high prevalence of ESBL- and carbapenemase-producing bacteria in hospital sewage and receiving rivers in China. These findings have serious implications for human health, and also suggest the need for more efforts to control the dissemination of resistant bacteria from hospital sewage into the environment.

## 1. Introduction

The emergence and rapid dissemination of antibiotic resistance is a serious and growing problem for human health [1]. β-lactam antibiotics are by far the most used antibiotics worldwide for treating infections in both humans and animals [2]. A significant threat to the usage of these agents is the rapid evolution of β-lactamases, mainly among Gram-negative bacteria, which makes each new drug obsolete in a very short period of time [3]. Among the β-lactams, carbapenem antibiotics are considered to be the most reliable last-resort treatment for bacterial infections caused by extended-spectrum β-lactamases (ESBLs)-producing bacteria [4]. The rapid spread of carbapenem resistance, usually caused by the production of carbapenemase, constitutes a critical public-healthcare problem worldwide [4]. Carbapenemases have the ability to hydrolyze penicillins, cephalosporins and carbapenems, thereby limiting treatment options [5]. Studies have shown an increased mortality associated with serious carbapenem-resistant *Enterobacteriaceae* (CRE) infections among hospitalized adults [6,7]. Various types of carbapenemases have been reported, among which the most commonly reported enzymes are the Ambler class A β-lactamases (e.g., KPC), the class B β-lactamases/metallo-β-lactamases (e.g., NDM and IMP) and the class D oxacillinases (e.g., OXA-48 and OXA-58) [8]. These carbapenemase genes are frequently located on mobile genetic elements and can be transferred among bacteria from different genera [9]. Particularly, bacteria belonging to the family *Enterobacteriaceae* and *Acinetobacter* usually function as important vectors in the dissemination of β-lactamase genes in natural bacterial ecosystems [10,11].

There is an increasing concern regarding the growing spread of antibiotic-resistant bacteria (ARB) in the environment. Sewage discharged from hospitals is a complex matrix with a collection of feces and urine of patients undergoing intensive antibiotic treatment and wastewater containing ARB from clinical settings. It serves as a reservoir of antibiotic resistance and a hotspot for horizontal gene transfer, enabling the spread of antibiotic resistance genes (ARGs) between bacterial communities [12]. Previous studies have identified a high abundance of ARB in hospital sewage around the world [13,14,15,16,17,18]. The first report concerning carbapenemases-producing bacteria (CPB) in hospital sewage in China in 2012 identified *bla*_KPC-2_-carrying *Citrobacter freundii* and *Enterobacter cloacae* in the influx of the wastewater treatment plants (WWTPs) [19]. After that, studies have detected a rich variety of resistant bacteria that produce carbapenemases, including NDM-1, KPC-2, and OXA-58 in hospital sewage in China [20,21,22,23]. Rivers are natural receptacles for large amounts of microbial contaminants from domestic sewage, livestock wastewater, and hospital effluents. Previous studies have also reported the isolation of CPB from water samples of rivers in different countries, such as KPC-2-producing *C. freundii* in China [24], OXA-48-like-producing *Escherichia coli* and *Klebsiella pneumoniae* in Algeria [25], and NDM-9-producing *Klebsiella variicola* in South Korea [26]. Studies by Azuma et al. and Khan et al. highlighted the dissemination of clinically significant ARB from hospitals into the receiving water bodies [27,28]. However, very limited studies have been conducted concerning the diversity and prevalence of ARGs and ARB in hospital sewage systems and receiving waters in China.

The aim of this study was, therefore, to (i) investigate the distribution and prevalence of carbapenemase- and ESBL-encoding genes from hospital sewage systems and receiving rivers in Southwest China, and (ii) characterize the pollution levels, with a focus on the *Enterobacteriaceae* and *Acinetobacter* isolates. Understanding the presence and spreading pathway of carbapenemase and ESBL genes in the hospital sewage and receiving waters are critical to assess their dissemination risk to public health through the aquatic environment.

## 2. Materials and Methods

### 2.1. Sample Collection

**Figure 1:**
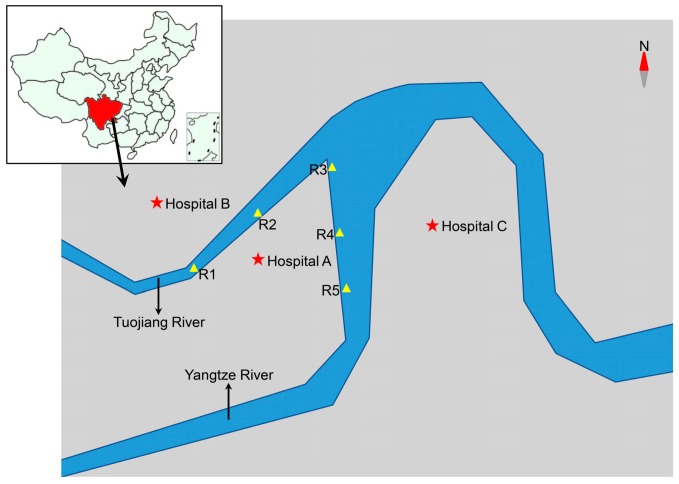
Schematic diagram of sampling sites of hospital sewage and river water, Luzhou City, Sichuan, China, 2019. The sampling sites of hospital sewage are indicated by stars. R1–R5: sampling sites of river water.

In August 2019, a total of six samples of hospital raw sewage and treated effluents were collected from hospital WWTPs of three tertiary care hospitals, with hospital A (2200 beds) being located in the center, hospital B (3000 beds) in the north, and hospital C (1000 beds) in the east of Luzhou City in Sichuan province, China. Five surface water samples were collected along receiving rivers influenced by these three hospitals. Each sampling site is at least 1 km apart (Figure 1). Water samples (500 mL) were collected during weekdays in the morning between 9.30 a.m. and 11 a.m. with sterile plastic bottles and taken to the laboratory for subsequent analysis within the following 1 h.

### 2.2. Isolation of ESBL- and Carbapenem-Resistant Gram-Negative Bacteria

Bacterial cells in water samples (6 mL for raw sewage, 120 mL for effluent samples and 200 mL for river water) were collected by centrifugation at 5000× *g* for 5 min, and resuspended in sterile 0.9% NaCl solution. Ten-fold serial dilutions (10–1000 times) of each water sample were made in sterile saline solution. A total of 100 μL of each dilution was then plated onto MacConkey agar supplemented with cefotaxime (4 μg/mL) or meropenem (2 μg/mL) for rapid screening of potential ESBL- or carbapenemase-producing Gram-negative bacteria. After incubated for 24 h at 37 °C, bacterial colonies with distinct coloration and morphologies were randomly picked and subcultured onto MacConkey agar containing cefotaxime (4 μg/mL) or meropenem (2 μg/mL) for further purification. A single colony of purified bacteria was picked and cultured in Trypticase Soy Broth. Each isolate was assigned a unique identification number and stored at −80 °C in 25% (vol/vol) glycerol for further investigation.

### 2.3. Bacterial Species Identification

Rapid bacterial genomic DNA extraction of selected isolates was performed by boiling method. Species identification was performed by PCR amplifying of the 16S rRNA gene using the primer pair 27F/1492R [29]. The PCR products were purified and then sequenced using Sanger sequencing by Tsingke Biotech (Beijing, China). Resulting nucleotide sequences of 16S rRNA genes were compared with sequences in GenBank (NCBI) database using BLAST software. 

### 2.4. Detection of Antibiotic Resistance Genes

All the isolates were examined for the presence of ten β-lactamase genes, including *bla*_CTX-M_, *bla*_KPC_, *bla*_NDM_, *bla*_OXA-48_, *bla*_OXA-58_, *bla*_IMP_, *bla*_VIM_ by PCR using specific primers (Table S1). All the PCR products were analyzed on 1.5% agarose gel by electrophoresis. Gene identification was further performed on all isolates positive for *bla*_CTX-M_ using primers *bla*_CTX-M-1 group_, *bla*_CTX-M-2 group_, *bla*_CTX-M-8 group_, and *bla*_CTX-M-9 group_. Sequences of *bla*_CTX-M-1/2/8/9 group_, *bla*_KPC_, and *bla*_NDM_ amplicons were determined using Sanger sequencing by Tsingke Biotech, and were compared with reported sequences available in the NCBI database using BLAST software.

### 2.5. Antimicrobial Susceptibility Testing

All *Enterobacteriaceae* isolates were evaluated for resistance to different antibiotic agents by the disk diffusion method according to the guidance of Clinical and Laboratory Standards Institute (CLSI) M100 [30]. The antibiotics tested were amoxicillin-clavulanic acid (AMC, 30 μg), ampicillin (AMP, 10 μg), ciprofloxacin (CIP, 5 μg), tetracycline (TET, 30 μg), gentamicin (CN, 10 μg), chloramphenicol (C, 30 μg), streptomycin (S, 10 μg), cefoxitin (FOX, 30 μg), cefotaxime (CTX, 30 μg), meropenem (MEM, 10 μg), and trimethoprim/sulfamethoxazole (SXT, 25 μg). In addition, all the *Acinetobacter* isolates were tested for susceptibility to CIP, TET, CN, FOX, CTX, MEM, and SXT using the disk diffusion method according to the CLSI. All the tested isolates were categorized as “S (sensitive)” or “R (resistant)” (Intermediate resistance, with inhibition zones larger than R and smaller than S, was excluded from the resistance percentage calculations). Mueller-Hinton agar (Solarbio, Beijing, China) was used for all assays, and zone diameters were determined following incubation at 37 °C for 16 to 24 h. Interpretation of zone diameters was done according to the CLSI criteria. *E. coli* ATCC 25,922 and *Pseudomonas aeruginosa* ATCC 27,853 were used as control in this study. The isolate was considered as multidrug-resistant (MDR), when it was resistant to three or more classes of antimicrobial agents.

## 3. Results

### 3.1. Bacterial Isolation

In the present study, a total of 104 non-repetitive Gram-negative bacteria were picked from MacConkey agar containing cefotaxime (n = 54, 51.9%) or meropenem (n = 50, 48.1%). Of them, 66 (63.5%) isolates were collected from raw sewage (hospital A, n = 20; hospital B, n = 20; hospital C, n = 26), 17 (16.3%) from effluent samples (hospital A, n = 3; hospital B, n = 2; hospital C, n = 12), and 21 (20.2%) were isolated from river water (Figure 1).

### 3.2. Bacterial Identification

A total of 62 (59.6%) out of the 104 isolates were found to be *Enterobacteriaceae*; 35/62 (56.5%) were *E. coli*, 17/62 (27.4%) *K. pneumoniae*, 5/62 (8.1%) *Enterobacter* spp., 3/62 (4.8%) *Citrobacter* spp., 2/62 (3.2%) *Raoultella planticola*, and 2/63 (3.2%) *K. variicola*. The remaining isolates were identified as: *Acinetobacter* spp. (20/104, 18.3%), *Aeromonas* spp. (11/104, 10.6%), *Pseudomonas* spp. (6/104, 5.8%), *Comamonas* spp. (2/104, 1.9%), *Stenotrophomonas maltophilia* (1/104, 0.96%), *Cupriavidus taiwanensis* (1/104, 0.96%) and *Proteus mirabilis* (1/104, 0.96%). 

Of the 66 isolates from raw sewage, the most predominant taxa were *E. coli* (25/66, 37.9%), *K. pneumoniae* (11/66, 16.7%), and *Acinetobacter* spp. (11/66, 16.7%). Resistant strains identified in effluent samples were dominated by *Pseudomonas* spp. (5/17, 29.4%), *Acinetobacter* spp. (5/17, 29.4%), and *E. coli* (4/17, 23.5%). The most commonly identified isolates from river water were *E. coli* (6/21, 28.6%), followed by *K. pneumoniae* (5/21, 23.8%) and *Acinetobacter* spp. (4/21, 19%). 

### 3.3. Detection and Molecular Characterization of Resistance Genes

**Figure 2:**
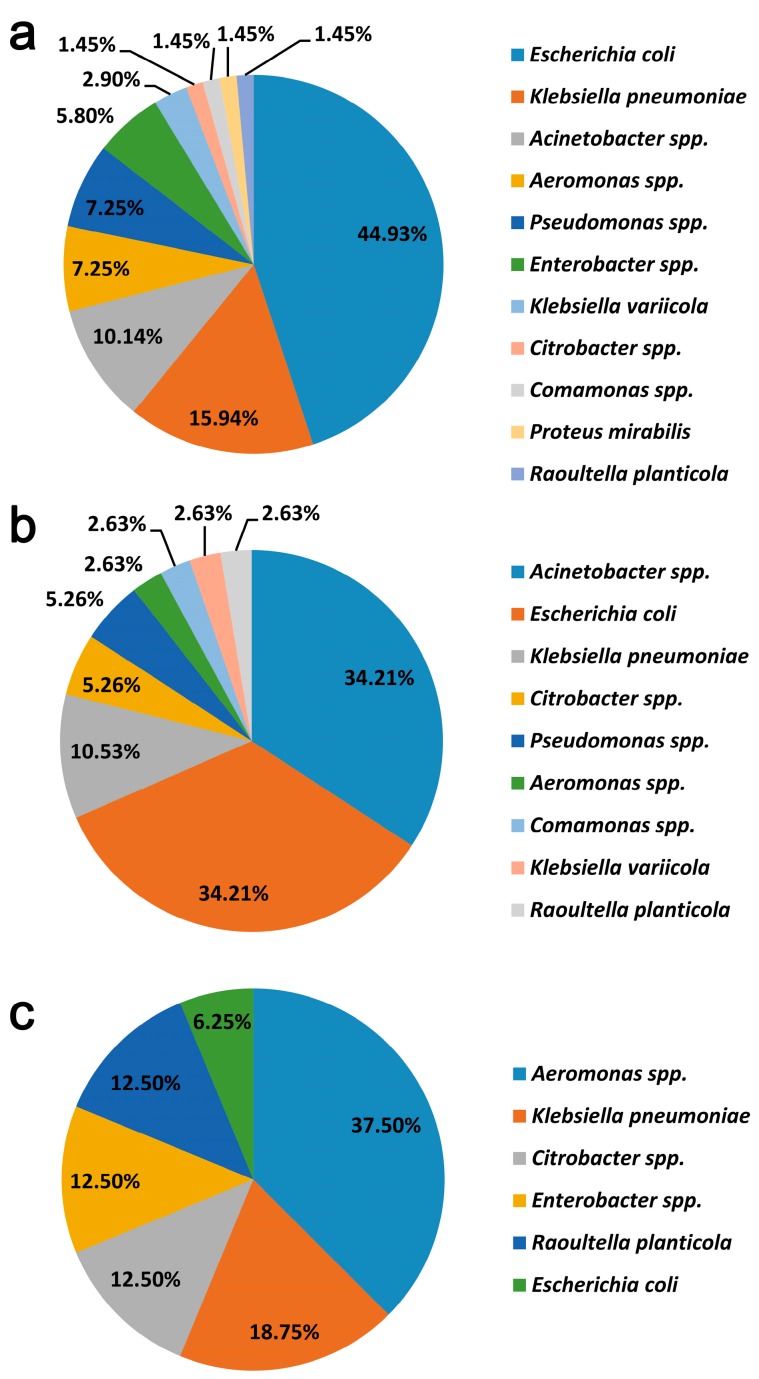
Distribution of the *bla*_CTX-M_ (**a**), *bla*_NDM_ (**b**), or *bla*_KPC_ (**c**) in the collected isolates in this study.

Of the 104 isolates, *bla*_CTX−M_-type gene was identified in a total of 69/104 (66.3%) strains. CTX-Ms were mainly harbored in species of *Enterobacteriaceae* (52/69, 75.4%), including *E. coli* (31/53), *K. pneumoniae* (11/53), *Enterobacter* spp. (4/53), *K. variicola* (2/53), *Citrobacter* spp. (1/53), and *R. planticola* (1/53) (Figure 2a). At least ten different CTX-M variants were identified in CTX-M producers, including CTX-M-55 (n = 31), CTX-M-3 (n = 7), CTX-M-14 (n = 6), CTX-M-15 (n = 5), CTX-M-65 (n = 4), CTX-M-27 (n = 3), CTX-M-199 (n = 2), CTX-M-123 (n = 2), CTX-M-9 (n = 1), and CTX-M-213 (n = 1). The subtypes of *bla*_CTX−M_ in seven isolates were not determined in this work.

The most frequently detected carbapenemase-encoding gene in this work was *bla*_NDM_ and it was present in 38/104 (36.5%) isolates (Figure 2b), of which 25 were recovered from raw sewage, 8 from effluent samples, and 5 from river water. *Bla*_NDM_ was frequently detected in species of *Enterobacteriaceae*, including *E. coli* (n = 13), *K. pneumoniae* (n = 4), and *Citrobacter spp*. (n = 2). It was noteworthy that a high detection rate of *bla*_NDM_ was also observed in *Acinetobacter* spp. (13/20, 65%). Sequencing analysis confirmed that the most frequently identified *bla*_NDM_ variant was *bla*_NDM-1_ (19/38, 50%), followed by *bla*_NDM-5_ (18/38, 47.4%). A *bla*_NDM-24_ gene was found to be present in one *Comamonas* sp. isolate from raw sewage. 

The second most prevalent carbapenemase-encoding gene was *bla*_KPC-2_, which was identified in 16/104 (15.4%) isolates (Figure 2c). A high proportion (n = 15, 93.8%) was isolated from raw sewage and that which remained was from an effluent sample. *Bla*_KPC-2_ was mostly identified in *Enterobacteriaceae*, including three *K. pneumoniae*, two *Enterobacter* spp., two *Citrobacter* spp., 2 *R. planticola*, *and* one *E. coli*. It was notable that *bla*_KPC-2_ was also detected in six *Aeromonas* strains. Additionally, *bla*_OXA-58_ was detected in seven (7/104, 6.7%) *Acinetobacter* isolates (five from raw sewage and two from effluent samples). One *Aeromonas* isolate (1/104, 0.96%) from raw sewage was found to harbor *bla*_IMP_ gene, which was further identified to be *bla*_IMP-4_ by whole genome sequence analysis of this strain (data not shown). None of the isolates were positive for OXA48-type or VIM-type carbapenemases. 

In addition, 28 out of 104 resistant strains (26.9%) coproduced CTX-M and carbapenemases, harbored by isolates of *Enterobacteriaceae* (n = 19), *Acinetobacter* spp. (n = 4), *Aeromonas* spp. (n = 3) and *Pseudomonas* spp. (n = 2). Of these resistant strains, 18 (64.3%) were recovered from raw sewage, nine (32.1%) from effluent samples and one (3.6%) from river water. The coexistence of NDM and OXA-58 was identified in seven *Acinetobacter* strains (five from raw sewage and two from effluent samples). Carbapenemase genes *bla*_KPC-2_ and *bla*_IMP-4_ were coharbored in an *Aeromonas* isolate from raw sewage. Additionally, two *Enterobacteriaceae* strains, one *Citrobacter* sp. and one *R. planticola* from raw sewage, were found to carry both *bla*_NDM-1_ and *bla*_KPC-2_ genes in addition to *bla*_CTX-M-14_.

### 3.4. Antimicrobial Susceptibility of Enterobacteriaceae and Acinetobacter Isolates

Analysis of the antimicrobial resistance of 62 *Enterobacteriaceae* strains (Table 1) revealed that all of them were resistant to ampicillin and cefotaxime. Furthermore, they presented a relatively high resistance to trimethoprim/sulfamethoxazole (77.4%) and tetracycline (74.2%), followed by amoxicillin-clavulanic acid (66.1%), cefoxitin (61.3%), and ciprofloxacin (61.3%). Medium resistance levels were found to gentamicin (51.6%), chloramphenicol (48.4%), and streptomycin (41.9%). A total of 32/62 (51.6%) *Enterobacteriaceae* strains were identified to be meropenem-resistant, of which 29 were positive for carbapenemase-encoding genes. Multidrug-resistant (MDR) strains, which were defined as resistance to at least 3 classes of antimicrobial agents, were commonly observed in the tested *Enterobacteriaceae* isolates by 85.5% (53/62). A total of 91.4% (32/35) *E*. *coli* and 94.1% (16/17) *K. pneumoniae* isolates were identified to be MDR. CRE strains isolated in this study showed a high percentage (28/32, 87.5%) of multi-drug resistance. It was noted that nine isolates demonstrated resistance to all 11 antimicrobial agents, including six NDM-5-producing *E. coli* (five from raw sewage and one from river water), two NDM-5-producing *K. pneumoniae* (one from effluent samples and the other from river water), and one NDM-5-producing *C. freundii* from river water.

Antibiotic susceptibility studies of *Acinetobacter* isolates revealed high levels of resistance to clinically important antibiotics (Table 2), including gentamicin (55%), cefoxitin (85%), cefotaxime (100%), tetracycline (75%), and trimethoprim/sulfamethoxazole (65%). However, *Acinetobacter* isolates showed a low resistance to ciprofloxacin (10%). It was worth noting that 14/20 (70%) *Acinetobacter* isolates showed resistance to meropenem, among which, 13 were found to carry at least one carbapenemase-encoding gene. Meanwhile, 75% (15/20) of *Acinetobacter* strains were found to be resistant to three or more antibiotic classes tested.

## 4. Discussion

Aquatic environments are significant reservoirs of antibiotic-resistant determinants. They also serve as a vehicle by which ARGs or ARB could be disseminated from one ecosystem to another, thereby increasing the risk of infection with MDR bacteria outside the hospital [31]. Among the MDR bacteria, the widespread nature of CPB posed a high risk to the global public health. In this work, CPB were readily detected in raw sewage, treated effluent and river water samples. NDM was identified to be the most prevalent carbapenemase and it was commonly harbored in *Enterobacteriaceae*, as well as in some non-*Enterobacteriaceae* families (*Acinetobacte*r spp., *Aeromonas* spp., and *Pseudomonas* spp.). Our results are consistent with previous studies, showing that *bla*_NDM_ has occurred in many unrelated species and is spreading rapidly in different environmental compartments [15,18,32]. The genus *Acinetobacter*, which is widespread in nature and particularly abundant in wastewater, has been recognized in recent years as a universal threat to public health [10]. The high prevalence of *bla*_NDM_ in *Acinetobacte*r species is worrisome, because these organisms may serve as potential reservoirs and propagate the *bla*_NDM_ gene through highly transmissible genetic elements, ultimately increasing the emergence of new population of carbapenem-resistant isolates. Multiple variants of *bla*_NDM_ have been identified in clinical and environmental samples [33]. Our results further supplement previous studies that show NDM-1 to be widely distributed in wastewater in China [20,34,35]. We also found that NDM-5, which exhibits increased resistance to carbapenems and broad-spectrum cephalosporins [33], was also highly prevalent in the collected samples. The wide dissemination of *bla*_NDM-5_ in the environment raises a serious public health concern.

KPC-producing isolates were frequently recovered from environmental samples in China [19,24,36,37]. *Aeromonas* spp. are among the dominant genera in wastewater communities and members of this genus are opportunistic pathogens of humans and animals [38]. Despite several studies reporting the emergence of multidrug-resistant and/or β-lactamase-producing *Aeromonas* isolates [38,39], a high prevalence of *bla*_KPC-2_ in *Aeromonas* in this study was still quite unexpected. A recent study also revealed two *Aeromonas* isolates carrying *bla*_KPC-2_ from urban wastewater treatment plant (WWTP) effluents in Japan [40]. The literature data and our findings suggest that *Aeromonas* spp. may be involved in the maintenance and dissemination of the *bla*_KPC-2_ gene in the environment.

The occurrence of strains co-producing two different carbapenemase families is increasingly frequent in environmental matrices. It was reported that *bla*_KPC_ and *bla*_NDM-1_ coexisted in two *P. aeruginosa* and one *C. freundii* from hospital effluents in Singapore [18]. One *Citrobacter braakii* strain was identified co-harboring *bla*_NDM-4_ and *bla*_OXA-48_ from hospital sewage in northern India [15]. An *Acinetobacter towneri* strain was identified co-harboring *bla*_NDM-1_ and *bla*_OXA-58_ from hospital sewage from one of our sampling sites (hospital A) [23]. In our work, the coexistence of *bla*_NDM-1_ and *bla*_OXA-58_ genes was identified in six *Acinetobacter* isolates, of which five were from hospital C and one from hospital B. Additionally, one *Acinetobacter* strain was found to co-harbor the *bla*_NDM-5_ and *bla*_OXA-58_ genes. These findings indicate that *Acinetobacter* strains coproducing NDM and OXA-58 were kind of prevalent in this region. The coexistence of NDM-1 and KPC-2 in environmental *Enterobacteriaceae* isolates, although not very common, was alarming and more research is needed to further elucidate the dissemination of these clinically significant antibiotic resistance genes.

Analysis of the antimicrobial resistance profiles of *Enterobacteriaceae* and *Acinetobacte*r isolates obtained in this study revealed a high prevalence of resistance to antimicrobial agents, and that MDR strains were frequently observed. In addition to β-lactam resistance, a relatively high percentage of resistance to trimethoprim/sulfamethoxazole and tetracycline was also identified. This finding was consistent with previous studies that tetracycline and sulfonamide resistance genes were widely found in aquaculture environments [15,41]. Notably, most carbapenem-resistant strains were cross-resistant to other classes of clinically important antibiotics, including quinolones (ciprofloxacin) and aminoglycosides (gentamicin). 

There is a growing number of studies reporting that, although WWTPs can efficiently remove many ARB, ARB or ARGs are still detectable at high concentrations in treated effluents [35,36,40]. In this study, we found that several carbapenemase-producing isolates persisted in the disinfected effluents from hospital WWTPs. We also detected carbapenemase producers in the receiving rivers which are affected by hospital WWTPs. These findings suggest that WWTP effluents could be a potential source for the dissemination of clinically important antibiotic resistance genes, such as carbapenemase genes, after their discharge into aquatic environments. The presence of a number of MDR opportunistic pathogens, such as *E*. *coli*, *K. pneumoniae*, *Enterobacter spp*. and *Acinetobacter spp*. in WWTP effluents and riverine environments represents an emerging public health issue. More efforts should be made to improve the elimination of antimicrobial resistant microorganisms from hospital sewage.

## 5. Conclusions

This study highlights the prevalence of ESBL- and carbapenemase-resistant *Enterobacteriaceae* and non-*Enterobacteriaceae* species (such as *Acinetobacter* and *Aeromonas*) in hospital sewage systems and receiving waters in China. Among these resistant isolates, a large proportion of the *Enterobacteriaceae* and *Acinetobacter* strains exhibited a multidrug resistance phenotype. The detection of clinically significant multidrug-resistant strains in the aquatic environment could have serious public health implications, which demands more attention. Furthermore, our findings also reinforce the idea that hospital WWTP effluent could be an important source of ARB propagation into the environment. Future projects should be conducted in more water bodies across the country to obtain a better picture of the situation. Superior disinfection techniques are expected to improve the elimination of ARB during wastewater treatment, and closer monitoring of the quality of treated effluents is needed to minimize the dissemination of antimicrobial resistance into the receiving environment.

## Figures and Tables

**Table 1:** *Enterobacteriaceae* isolated from aquatic environments.

Isolation No. ^a^	Species	Isolation Source	AMP ^b^	CTX	FOX	MEM	AMC	CN	S	C	TET	CIP	SXT	NDM	KPC	CTX-M
FA8C	*Klebsiella pneumoniae*	hospital A/raw sewage	R	R	S	S	R	R	R	R	R	R	R			3
FA9C	*Escherichia coli*	hospital A/raw sewage	R	R	S	S	S	S	S	S	S	R	S			3
FA11C	*Klebsiella pneumoniae*	hospital A/raw sewage	R	R	S	S	I	I	S	S	R	R	R			15
FA1C	*Escherichia coli*	hospital A/raw sewage	R	R	R	S	I	S	R	R	R	R	R			123
FA3C	*Klebsiella pneumoniae*	hospital A/raw sewage	R	R	S	S	I	R	R	R	R	R	R			15
FA4C	*Escherichia coli*	hospital A/raw sewage	R	R	R	S	R	R	I	R	R	R	R			15
FA6C	*Enterobacter* sp.	hospital A/raw sewage	R	R	R	S	R	S	S	S	S	S	S			65
FA7C	*Escherichia coli*	hospital A/raw sewage	R	R	S	S	S	S	R	R	R	S	R			55
FA3M	*Klebsiella pneumoniae*	hospital A/raw sewage	R	R	R	R	R	S	I	R	R	S	R	5		
FA5M	*Klebsiella pneumoniae*	hospital A/raw sewage	R	R	R	R	R	S	I	R	R	S	R	5		
FA7M	*Raoultella planticola*	hospital A/raw sewage	R	R	R	R	R	R	S	S	R	R	R	1	2	14
FA9M	*Escherichia coli*	hospital A/raw sewage	R	R	R	R	R	R	R	R	R	R	R	5		55
FA4M	*Citrobacter braakii*	hospital A/raw sewage	R	R	R	R	R	R	S	S	R	R	R		2	
ZA4M	*Escherichia coli*	hospital B/raw sewage	R	R	R	R	R	S	S	I	R	R	R	5		14
ZA3M	*Escherichia coli*	hospital B/raw sewage	R	R	R	R	I	S	S	S	S	R	R	5		15
ZA1M	*Escherichia coli*	hospital B/raw sewage	R	R	R	R	R	R	R	R	R	R	R	5		199
ZA10M	*Escherichia coli*	hospital B/raw sewage	R	R	R	R	R	R	R	R	R	R	R	5		199
ZA9M	*Klebsiella pneumoniae*	hospital B/raw sewage	R	R	R	R	R	S	R	R	R	R	R			27
ZA7M	*Citrobacter* sp.	hospital B/raw sewage	R	R	R	R	R	R	S	S	R	R	R	1	2	14
ZA5M	*Escherichia coli*	hospital B/raw sewage	R	R	R	R	R	R	S	S	S	R	R	5		123
ZA7C	*Klebsiella pneumoniae*	hospital B/raw sewage	R	R	R	S	R	S	S	S	R	S	R			
ZA1C	*Escherichia coli*	hospital B/raw sewage	R	R	S	S	S	R	R	S	R	S	R			55
ZA2C	*Escherichia coli*	hospital B/raw sewage	R	R	R	S	I	S	I	S	S	R	R			55
ZA4C	*Escherichia coli*	hospital B/raw sewage	R	R	R	S	R	R	R	R	R	I	S			55
ZA5C	*Escherichia coli*	hospital B/raw sewage	R	R	S	S	S	R	R	S	R	S	R			55
ZA6C	*Escherichia coli*	hospital B/raw sewage	R	R	S	S	I	R	S	S	R	R	R			55
RA2M	*Escherichia coli*	hospital C/raw sewage	R	R	R	R	R	R	R	R	R	R	R	5		55
RA1M	*Enterobacter kobei*	hospital C/raw sewage	R	R	R	R	R	S	S	S	S	S	S		2	9
RA13C	*Raoultella planticola*	hospital C/raw sewage	R	R	S	R	R	R	S	S	S	S	R		2	
RA14M	*Klebsiella pneumoniae*	hospital C/raw sewage	R	R	R	R	R	S	I	R	R	R	S		2	
RA11M	*Klebsiella pneumoniae*	hospital C/raw sewage	R	R	R	R	R	R	S	R	S	R	R		2	65
RA6C	*Klebsiella pneumoniae*	hospital C/raw sewage	R	R	R	R	R	R	S	S	S	S	R		2	
RA5C	*Escherichia coli*	hospital C/raw sewage	R	R	S	S	I	S	R	R	R	S	R			55
RA4C	*Escherichia coli*	hospital C/raw sewage	R	R	S	R	I	R	I	S	R	R	R	1		14
RA2C	*Escherichia coli*	hospital C/raw sewage	R	R	S	S	R	S	R	I	R	R	R			55
RA1C	*Escherichia coli*	hospital C/raw sewage	R	R	S	S	R	S	I	R	R	S	S			55
RA10M	*Enterobacter* sp.	hospital C/raw sewage	R	R	R	R	R	S	S	S	S	S	S		2	55
RA7M	*Escherichia coli*	hospital C/raw sewage	R	R	R	R	R	R	R	R	R	R	R	5		+
RA12C	*Escherichia coli*	hospital C/raw sewage	R	R	S	R	R	S	S	S	S	R	S	1		55
RA11C	*Escherichia coli*	hospital C/raw sewage	R	R	S	R	S	S	S	S	S	R	S	1		
RA8C	*Klebsiella pneumoniae*	hospital C/raw sewage	R	R	R	S	R	R	S	R	R	R	R			27
RA7C	*Escherichia coli*	hospital C/raw sewage	R	R	S	S	R	R	S	S	R	R	R			15
RA8M	*Escherichia coli*	hospital C/raw sewage	R	R	R	R	R	R	R	R	R	S	R			55
RB1C	*Escherichia coli*	hospital C/effluent	R	R	S	S	I	R	R	R	R	R	R			55
RB7C	*Escherichia coli*	hospital C/effluent	R	R	I	R	S	S	I	S	S	R	R		2	14
RB6C	*Escherichia coli*	hospital C/effluent	R	R	S	S	I	R	R	R	R	R	R			55
RB4C	*Escherichia coli*	hospital C/effluent	R	R	S	S	R	S	S	R	R	S	S			55
RB3M	*Klebsiella pneumoniae*	hospital C/effluent	R	R	R	R	R	R	R	R	R	R	R	5		55
RB6M	*Klebsiella variicola*	hospital C/effluent	R	R	R	R	R	R	R	R	R	S	R	5		55
CS3C	*Escherichia coli*	river water	R	R	S	S	S	S	S	S	S	R	R			14
CS4C	*Enterobacter tabaci*	river water	R	R	R	S	R	S	S	S	S	S	S			+
CS8C	*Escherichia coli*	river water	R	R	S	S	R	S	R	S	R	R	R			55
TX1C	*Klebsiella variicola*	river water	R	R	R	S	R	S	S	S	R	S	R			+
TX2C	*Klebsiella pneumoniae*	river water	R	R	S	S	S	S	S	R	R	S	R			27
TX5C	*Klebsiella pneumoniae*	river water	R	R	R	S	R	S	R	S	R	S	R			+
TS1C	*Klebsiella pneumoniae*	river water	R	R	R	S	S	S	S	S	S	S	S			+
TS11C	*Escherichia coli*	river water	R	R	S	S	S	S	S	S	R	S	S			
TS3M	*Escherichia coli*	river water	R	R	R	R	R	R	R	R	R	R	R	5		
TS4M	*Klebsiella pneumoniae*	river water	R	R	R	R	R	R	R	R	R	R	R	5		
CX1M	*Escherichia coli*	river water	R	R	R	R	R	R	R	S	R	R	S	1		
TX5M	*Citrobacter freundii*	river water	R	R	R	R	R	R	R	R	R	R	R	5		
TX6M	*Escherichia coli*	river water	R	R	R	R	R	R	I	R	R	S	R			

^a^ Multidrug-resistant strains are highlighted in orange. ^b^ ampicillin (AMP), amoxicillin-clavulanic acid (AMC), ciprofloxacin (CIP), tetracycline (TET), gentamicin (CN), chloramphenicol (C), streptomycin (S), cefoxitin (FOX), cefotaxime (CTX), meropenem (MEM), and trimethoprim/sulfamethoxazole (SXT). Antibiotic susceptibility is depicted with S for susceptible, I for intermediate resistant and R for resistant (highlighted in green).

**Table 2:** *Acinetobacter* strains isolated from aquatic environments.

Isolation No. ^a^	Isolation Source	CTX ^b^	FOX	MEM	CN	TET	CIP	SXT	NDM	OXA	CTX-M
FA2C	hospital A/raw sewage	R	R	S	S	S	S	S			65
FA2M	hospital A/raw sewage	R	R	R	R	R	R	R	1		
ZA2M	hospital B/raw sewage	R	R	R	R	R	I	R	1	58	
ZA6M	hospital B/raw sewage	R	R	R	R	R	S	R	1		
ZA8M	hospital B/raw sewage	R	S	S	R	S	S	I			
ZA8C	hospital B/raw sewage	R	R	S	S	S	S	S			55
RA5M	hospital C/raw sewage	R	R	R	S	R	S	R	1		
RA9M	hospital C/raw sewage	R	R	R	R	R	R	R	1	58	
RA12M	hospital C/raw sewage	R	R	R	R	R	I	R	5	58	3
RA3M	hospital C/raw sewage	R	R	R	S	R	I	R	1	58	
RA4M	hospital C/raw sewage	R	R	R	R	R	I	R	1	58	
RB4M	hospital C/effluent	R	R	R	R	R	S	R	1		
RB5C	hospital C/effluent	R	R	R	R	R	S	R	1		55
RB1M	hospital C/effluent	R	R	R	R	R	I	R			55
RB2M	hospital C/effluent	R	R	R	R	R	S	R	1	58	55
RB5M	hospital C/effluent	R	R	R	S	R	I	I	1	58	3
CX9C	river water	R	I	S	S	S	S	S			
CX11C	river water	R	S	S	S	R	S	S			
TX3C	river water	R	R	S	S	R	S	R			
CS1M	river water	R	R	R	S	S	S	S	1		

^a^ Multidrug-resistant strains are highlighted in orange. ^b^ cefotaxime (CTX), cefoxitin (FOX), meropenem (MEM), gentamicin (CN), tetracycline (TET), ciprofloxacin (CIP), and trimethoprim/sulfamethoxazole (SXT). Antibiotic susceptibility is depicted with S for susceptible, I for intermediate resistant and R for resistant (highlighted in green).

## Supplementary Materials

The following are available online at https://www.mdpi.com/1660-4601/17/4/1183/s1, Table S1: Primers used in this study.
