# Supplementary material for: The Prevalence and Characterization of Extended-Spectrum β-Lactamase- and Carbapenemase-Producing Bacteria from Hospital Sewage, Treated Effluents and Receiving Rivers
Source: Int J Environ Res Public Health. 2020 Feb 13;17(4):1183. doi: 10.3390/ijerph17041183 (PMC7068339; doi:10.3390/ijerph17041183)
Supplement: Supplementary file 1 [file ijerph-17-01183-s001.pdf]

Table S1 Primers used in this study.

Primer name	Sequence (5'-3')	Reference
27F	AGAGTTTGATCCTGGCTCAG	(Lane, 1991)
1492R	ACGGCTACCTTGTTACGACTT	
bla_{CTX-M}-F	CGATGTGCAGTACCAGTAA	(Batchelor et al., 2005)
bla_{CTX-M}-R	TTAGTGACCAGAATCAGCGG	
$bla_{CTX-M-1\ group}$ -F	CAGCGCTTTTGCCGTCTAAG	(Yu et al., 2007)
$bla_{CTX-M-1\ group}$ -R	GGCCCATGGTTAAAAAATCACTGC	
$bla_{CTX-M-2\ group}$ -F	CTCAGAGCATTCGCCGCTCA	(Yu et al., 2007)
$bla_{CTX-M-2\ group}$ -R	CCGCCGCAGCCAGAATATCC	
$bla_{CTX-M-8\ group}$ -F	ACTTCAGCCACACGGATTCA	(Yu et al., 2007)
$bla_{CTX-M-8\ group}$ -R	CGAGTACGTCACGACGACTT	
$bla_{CTX-M-9\ group}$ -F	GTTACAGCCCTTCGGCGATGATTC	(Yu et al., 2007)
$bla_{CTX-M-9\ group}$ -R	GCGCATGGTGACAAAGAGAGTGCAA	
bla_{VIM}-F	GATGGTGTTTGGTCGCATA	(Ellington et al., 2007)
bla_{VIM}-R	CGAATGCGCAGCACCAG	
bla_{KPC}-F (full)	CGCTACACCTAGCTCCACCTTC	This study
bla_{KPC}-R (full)	CGGTGGTGGGCCAATAGATG	
bla_{NDM}-F (full)	ATTTACTAGGCCTCGCATTTGC	This study
bla_{NDM}-R (full)	GCCTCTGTCACATCGAAATCG	
bla_{IMP}-F	GGAATAGAGTGGCTTAAYTCTC	(Ellington et al., 2007)
bla_{IMP}-R	CCAAACYACTASGTTATCT	
bla_{OXA-48}-F	TTGGTGGCATCGATTATCGG	(Szekely et al., 2013)
bla_{OXA-48}-R	GAGCACTTCTTTTGTGATGGC	
bla_{OXA-58}-F	AAGTATTGGGGCTTGTGCTG	(Asadollahi et al., 2012)
bla_{OXA-58}-R	CCCCTCTGCGCTCTACATAC	

References

Asadollahi, P, Akbari, M, Soroush, S, Taherikalani, M, Asadollahi, K, Sayehmiri, K, Maleki, A, Maleki, M H, Karimi, P, Emaneini, M, 2012. Antimicrobial resistance patterns and their encoding genes among *Acinetobacter baumannii* strains isolated from burned patients. Burns 38:1198-1203.
Batchelor, M, Hopkins, K, Threlfall, E J, Clifton-Hadley, F A, Stallwood, A D, Davies, R H, Liebana, E, 2005. *bla*(CTX-M) genes in clinical *Salmonella* isolates recovered from humans in England and Wales from 1992 to 2003. Antimicrob Agents Chemother 49:1319-1322.
Ellington, M J, Kistler, J, Livermore, D M, Woodford, N, 2007. Multiplex PCR for rapid detection of genes encoding acquired metallo-beta-lactamases. J Antimicrob Chemother 59:321-322.
Lane, D J, 1991. 16S/23S rRNA sequencing. Nucleic acid techniques in bacterial systematics:115-175.
Szekely, E, Damjanova, I, Janvari, L, Vas, K E, Molnar, S, Bilca, D V, Lorinczi, L K, Toth, A, 2013. First description of *bla*(NDM-1), *bla*(OXA-48), *bla*(OXA-181) producing *Enterobacteriaceae* strains in Romania. Int J Med Microbiol 303:697-700.
Yu, Y, Ji, S, Chen, Y, Zhou, W, Wei, Z, Li, L, Ma, Y, 2007. Resistance of strains producing extended-spectrum beta-lactamases and genotype distribution in China. The Journal of infection

54:53-57.
